# A nomogram based on radiomics intermuscular adipose analysis to indicate arteriosclerosis in patients with newly diagnosed type 2 diabetes

**DOI:** 10.3389/fendo.2023.1201110

**Published:** 2023-05-26

**Authors:** Cong He, Dong Xie, Lin-feng Fu, Jin-na Yu, Fang-ye Wu, Yong-gang Qiu, Hong-wei Xu

**Affiliations:** Department of Radiology, Shaoxing Second Hospital, Shaoxing, Zhejiang, China

**Keywords:** radiomics, nomogram, arteriosclerosis, newly diagnosed diabetes, CT

## Abstract

**Objective:**

Early identifying arteriosclerosis in newly diagnosed type 2 diabetes (T2D) patients could contribute to choosing proper subjects for early prevention. Here, we aimed to investigate whether radiomic intermuscular adipose tissue (IMAT) analysis could be used as a novel marker to indicate arteriosclerosis in newly diagnosed T2D patients.

**Methods:**

A total of 549 patients with newly diagnosed T2D were included in this study. The clinical information of the patients was recorded and the carotid plaque burden was used to indicate arteriosclerosis. Three models were constructed to evaluate the risk of arteriosclerosis: a clinical model, a radiomics model (a model based on IMAT analysis proceeded on chest CT images), and a clinical-radiomics combined model (a model that integrated clinical-radiological features). The performance of the three models were compared using the area under the curve (AUC) and DeLong test. Nomograms were constructed to indicate arteriosclerosis presence and severity. Calibration curves and decision curves were plotted to evaluate the clinical benefit of using the optimal model.

**Results:**

The AUC for indicating arteriosclerosis of the clinical-radiomics combined model was higher than that of the clinical model [0.934 (0.909, 0.959) vs. 0.687 (0.634, 0.730), *P* < 0.001 in the training set, 0.933 (0.898, 0.969) vs. 0.721 (0.642, 0.799), *P* < 0.001 in the validation set]. Similar indicative efficacies were found between the clinical-radiomics combined model and radiomics model (*P* = 0.5694). The AUC for indicating the severity of arteriosclerosis of the combined clinical-radiomics model was higher than that of both the clinical model and radiomics model [0.824 (0.765, 0.882) vs. 0.755 (0.683, 0.826) and 0.734 (0.663, 0.805), *P* < 0.001 in the training set, 0.717 (0.604, 0.830) vs. 0.620 (0.490, 0.750) and 0.698 (0.582, 0.814), *P* < 0.001 in the validation set, respectively]. The decision curve showed that the clinical-radiomics combined model and radiomics model indicated a better performance than the clinical model in indicating arteriosclerosis. However, in indicating severe arteriosclerosis, the clinical-radiomics combined model had higher efficacy than the other two models.

**Conclusion:**

Radiomics IMAT analysis could be a novel marker for indicating arteriosclerosis in patients with newly diagnosed T2D. The constructed nomograms provide a quantitative and intuitive way to assess the risk of arteriosclerosis, which may help clinicians comprehensively analyse radiomics characteristics and clinical risk factors more confidently.

## Introduction

Diabetes is one of the major conditions that endangers human health worldwide (1). Evidence has confirmed that cardiovascular and cerebrovascular accidents are the major outcomes of individuals with diabetes, whose pathological changes are characterized by arteriosclerosis (2). Even in the state of newly diagnosed diabetes, the risk of arteriosclerosis is present (3). Evaluating the severity of arteriosclerosis can predict the risk of cardio-cerebral vascular events, such as stroke, myocardial infarction, and amputation, in patients with both newly diagnosed and known diabetes (4).

It has been reported that before the onset of T2D, insulin resistance (IR) is generally present and acts as an independent risk factor for the development of arteriosclerosis (5). Several indicators can reflect IR, such as the hyperinsulinaemic euglycaemic clamp (HEC), insulin resistance index (HOMA-IR), triglyceride-glucose index (TyG), triglyceride/high-density lipoprotein cholesterol (TG/HDL-C), visceral fat index (VAI) (6–8). The HEC is the gold standard for IR measurement. However, it is time-consuming and requires frequent blood collection, which limits its clinical application. Other blood examination indicators, such as HOMA-IR, TyG, and TG/HDL-C, may have limited sensitivity and specificity (9).

Intermuscular adipose tissue (IMAT) is a kind of distinct adipose that accumulates within the skeletal muscle and the content of IMAT has been shown to be related to IR in recent studies (10–12). Using imaging modalities, such as CT and MRI, noninvasive quantification of IMAT can be performed for diabetes management (11, 13). However, *in vivo* quantitative analysis of IMAT relies on image segmentation techniques or thresholding. It is operator-dependent and may lack accuracy and robustness (14, 15). Radiomics is an emerging approach that makes quantitative assessment of medical features extracted from a region of interest possible by mathematical-statistical algorithms (16). It has been used to explore and model the association between features and survival or malignancy prediction (17, 18). However, the relationship between radiomics intermuscular adipose analysis and arteriosclerosis in patients with newly diagnosed type 2 diabetes (T2D) is scarce to date.

Therefore, our study aimed to investigate the association of radiomic features for IMAT analysis with arteriosclerosis in newly diagnosed T2D patients. Moreover, we developed nomogram prediction models based on radiomics IMAT assessment and clinical risk factors to indicate arteriosclerosis and compared whether the integration of these methods enhances the indication performance.

## Materials and methods

### Study participants in the study

**Figure 1:**
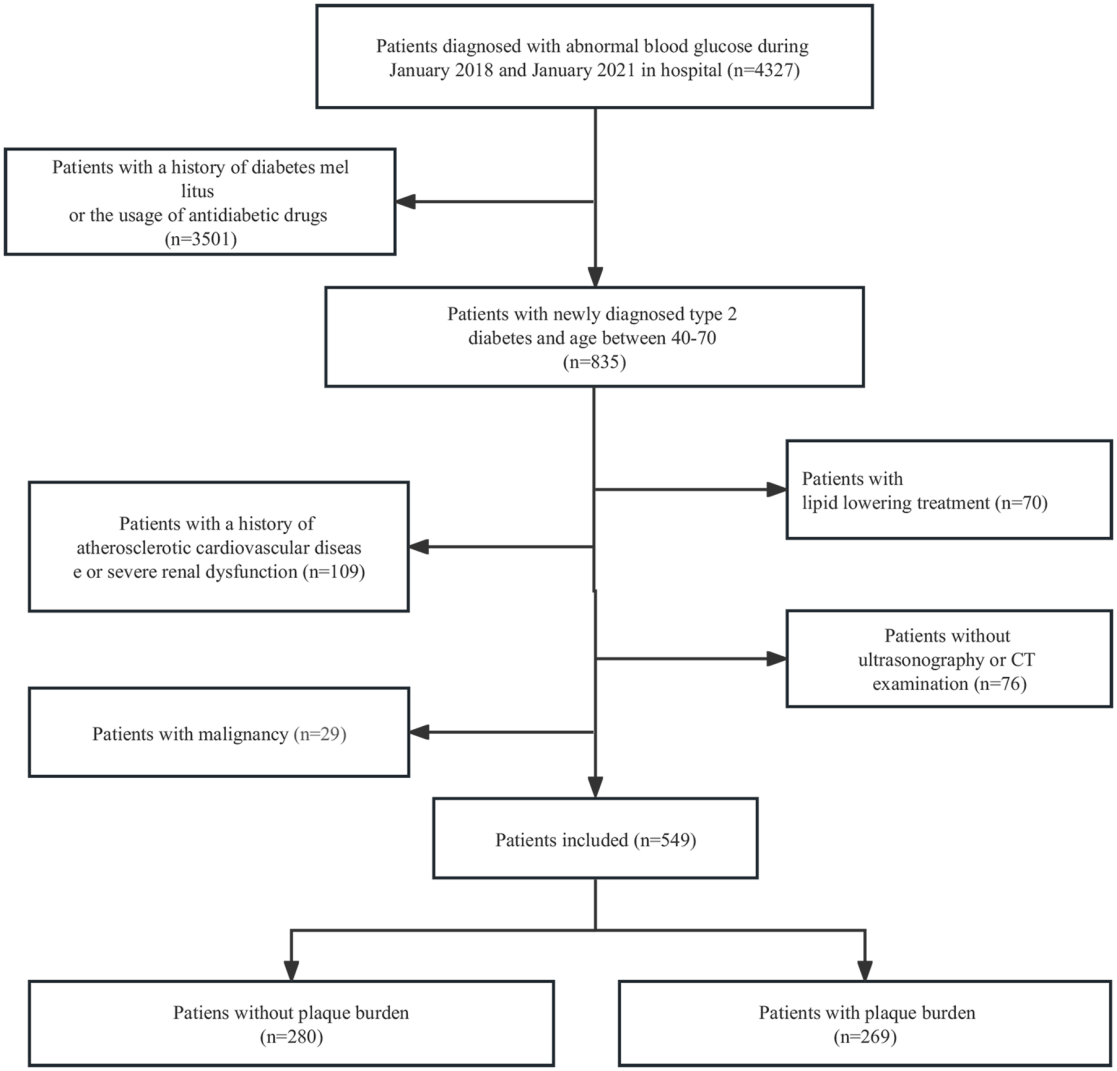
Inclusion and exclusion criteria of our study.

This study was approved by the Ethics Committee of Shaoxing Second Hospital. The data were anonymous, so informed consent was waived. From January 2018 to January 2021, data from 4327 hospitalized patients with abnormal blood glucose levels were retrospectively analysed. After reviewing clinical information, a total of 549 patients with newly diagnosed T2D were included in the study. The inclusion criteria were as follows: 1) fasting plasma glucose (FPG) ≥ 7.0 mmol/l, 2-hour postprandial glucose (2hPG) ≥ 11.1 mmol/l, and/or glycated haemoglobin (HbA1c) ≥ 6.5% (19); 2) abnormal blood glucose duration ≤ six months; and 3) age between 40 and 70 years old. The exclusion criteria were as follows: 1) history of antidiabetic drug use; 2) history of lipid-lowering treatment; 3) history of arteriosclerotic cardiovascular disease or severe renal dysfunction; 4) history of malignancy; and 5) lack of carotid ultrasonography or chest CT examination. Arteriosclerotic cardiovascular disease includes stroke, transient ischemic attack, coronary heart disease, heart failure, and arterial occlusion (3). We randomly divided the patients into a training set and a validation set at a ratio of 7:3. The details of patient selection are presented in **Figure 1**.

### Data collection

Data on sex, age, height, weight, waist circumference, tobacco use, alcohol use and history of hypertension were collected. Body mass index (BMI) was calculated as weight (kg) divided by the squared value of height (m^2^). Hypertension was defined as blood pressure (BP) ≥140/90 mmHg on two different occasions or a history of antihypertensive treatment.

Clinical laboratory test data, such as triglycerides (TGs), total cholesterol (TC), triglycerides/high-density lipoprotein cholesterol (TG/HDL-C), FPG, and HbA1c, were also recorded. The TyG index was calculated by the equation log [(fasting TG (mg/dl) × FPG (mg/dl)/2] (20).

### Assessment of arteriosclerosis

The extent of arteriosclerosis in the participants was assessed by carotid ultrasonography according to the literature (3). Briefly, for each participant, the unilateral carotid artery was divided into the common carotid region, internal carotid region, external carotid region, and bifurcation carotid region. The presence of plaques in each region was scored as 1 (single plaque), 2 (multiple plaques), or 3 (stenosis). The plaque score (PS) was calculated by summing the scores of both carotid arteries. Based on the PS, the severity of arteriosclerosis was classified into no plaque burden (PS = 0), low plaque burden (PS < 3), and high plaque burden (PS ≥ 3). The ultrasound images were independently reviewed by two experienced sonographers who had no knowledge of the clinical data. Any discrepancies were resolved by consensus.

### Radiomics IMAT analysis

Radiomics IMAT analysis was proceeded on chest CT images obtained by using a 64-row or 16-row multidetector CT scanner (SOMATOM Definition AS, Siemens Medical Solutions, and BrightSpeed, GE Healthcare). The following CT parameters were applied: rotation time, 500 milliseconds; voltage, 120 kVp; automatic exposure control, and 2.5 mm reconstructed section thickness.

The acquired CT images were then imported into ITK-SNAP software (www.itksnap.org) to delineate the regions of interest (ROIs) of IMAT in the section above the aortic arch by two independent experienced radiologists who had no knowledge of the arteriosclerosis degree (13). To ensure the consistency of the data, the same delineate criterion was applied, and another expert was responsible for conforming the segmentation. After image segmentation, Z score normalization was applied to standardize the CT images. To assess the reproducibility of image segmentation, two months later, 50 patients were randomly selected, and the IMAT ROIs were delineated again by radiologists to build a resegmentation set. Then, intraobserver and interobserver repeatability were evaluated by using intraclass and interclass correlation coefficients (ICCs).

After the image segmentation, the radiomics features of IMAT were extracted with the pyradiomic platform (https://keyan.deepwise.com) (21). In the preprocessing stage, Z score normalization was used to process the images with a normalization scale of 100, and the B-spline interpolation sampling method was used to resample CT images to the same resolution. Eight kinds of filters (wavelet, Laplacian of Gaussian, square, square root, logarithm, exponential, gradient transform, and local binary pattern transform) were applied to preprocess the CT images. In total, 1,316 radiomics features of IMAT were extracted from the ROIs of each CT image: 252 first-order features, 14 shape features, 336 greyscale co-occurrence matrix (GLCM), 224 grey-level size zone matrix (GLSZM), 224 grey-level run length matrix (GLRLM), 196 grey-level distance-zone matrix (GLDM), and 70 neighbourhood grey-tone difference matrix (NGTDM).

Least absolute shrinkage and selection operator (LASSO) regression was used to select the extracted radiomic features of IMAT that were highly correlated with arteriosclerosis. First, 260 features with unique values were excluded. *P*earson correlation analysis was used to estimate the correlation between the remaining features. Features with a correlation coefficient under 0.90 were excluded. After dimensionality reduction of features, the 144 features were included in subsequent modelling.

### Model construction and validation

Both clinical data and radiomics features were applied for model construction in indicating arteriosclerosis in patients with newly diagnosed T2D. In terms of clinical elements, univariate and multivariate logistic regression were conducted to explore the relationship between clinical elements and the carotid plaque burden, which represented arteriosclerosis. Then, clinical elements with *P* < 0.05 in multivariate analysis were included to establish model 1. With regard to the radiomics model, the L1-based method was used for IMAT radiomics feature selection. By summing the included features weighted by their coefficients, a radiomic score (Rad-score) formula (model 2) could be constructed. A combined clinical-radiomics model (model 3) was developed by integrating the clinical elements and the radiomics signature and presented in the form of a nomogram.

**Figure 2:**
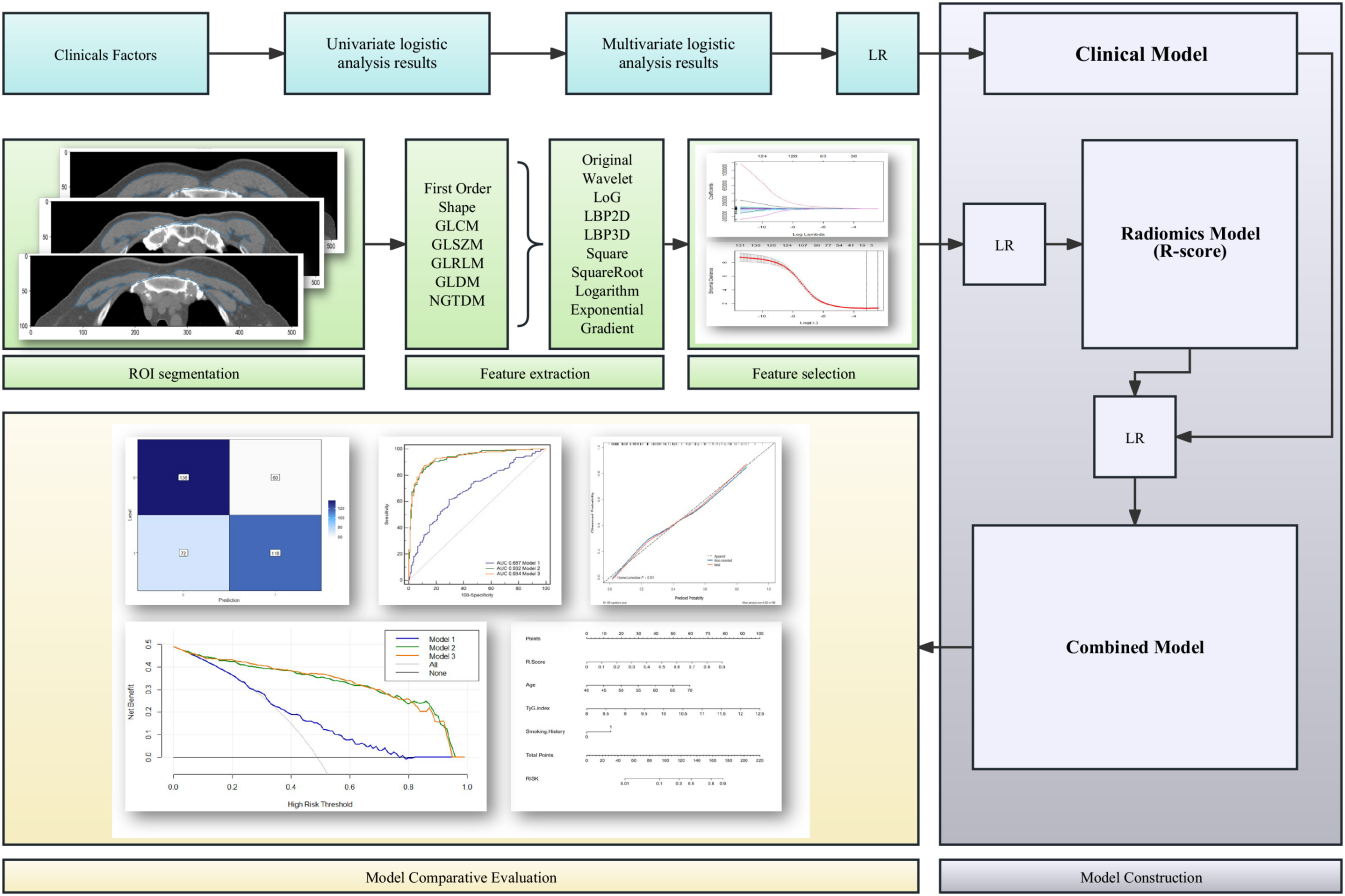
Flowchart of the development and evaluation of the clinical model, radiomics model, and clinical-radiomics combined model. LR, linear regression; ROI, regions of interest; GLCM, co-occurrence matrix; GLSZM, grey-level size zone matrix; GLRLM, grey-level run length matrix; GLDM, grey-level distance-zone matrix; NGTDM, neighbourhood grey-tone difference matrix.

A confusion matrix was used to quantify the performances of model 1, model 2, and model 3. Furthermore, receiver operating characteristic (ROC) curves were generated to quantify the clinical usefulness of the three models. Calibration curves were plotted to determine the discrimination ability of the radiomic-clinical nomogram for the training and validation sets. The usefulness of the radiomic-clinical nomogram was assessed by the net benefits in different threshold probabilities by decision curve analysis (DCA). **Figure 2** presents the flowchart of the study.

### Statistical analysis

All statistical analyses were performed with SPSS (version 26.0), MedCalc (vision 19.5.6), and R software (version 4.0.2). Normality was evaluated by the Kolmogorov-Smirnov test. Data with a nonnormal distribution were expressed as medians (interquartile ranges) for continuous variables and as percentages for categorical variables. Group differences were evaluated by the Wilcoxon rank sum test for continuous variables and by the chi-square test for categorical variables. Pearson’s chi-squared test was used to identify the difference between the training and validation sets. The performance of the clinical model, radiomics model, and combined clinical-radiomics model was quantified by the area under the curve (AUC) with 95% confidence intervals (95% CIs). The DeLong test was employed to determine whether significant differences existed in the AUC values of the three models. We also calculated the ICCs to evaluate the agreement of extracted radiomics features by two radiologists. Kappa test analyses were used to determine the intra- and interobserver agreement. *P* values ≤ 0.05 were considered statistically significant.

## Results

### Clinical characteristics of the patients

The baseline characteristics of the patients with or without plaque burden are compared in **Table 1**. Patients with plaque burden were older, more likely to be male, had more hypertension, and were more likely to be current smokers (57.00 [52.00-63.00] vs. 52.00 [46.00-58.00)] years, *P* < 0.001; 70.60% vs. 61.80%, *P* =0.029; 51.70% vs. 36.80%, *P* < 0.001; 49.80% vs. 37.10%, *P* =0.003, respectively). However, there were no differences in the physical examination results (such as BMI and waist circumference) and laboratory findings (such as TGs, TC, FPG, and the TyG index) between the groups.

**Table 1:** Baseline characteristics of all subjects (n=549).

	Without plaque burden (n=280)	With plaque burden (n=269)	*P*-value
Age (years)	52.00 (46.00-58.00)	57.00 (52.00-63.00)	<0.001^*^
Men (n, %)	173 (61.80)	190 (70.60)	0.029^*^
BMI (kg/m^2^)	24.80 (22.68-26.60)	24.50 (22.44-27.10)	0.700
Waist circumference (cm)	88.00 (83.00-95.00)	89.00 (81.00-94.00)	0.581
SBP (mmHg)	129.00 (118.00-140.00)	136.00 (123.00-148.00)	<0.001^*^
DBP (mmHg)	83.00 (76.00-90.00)	84.00 (76.00-90.00)	0.556
Hypertension (n, %)	103 (36.80)	139 (51.70)	<0.001^*^
Current smoking (n, %)	104 (37.10)	134 (49.80)	0.003^*^
Alcohol consumption (n, %)	90 (32.10)	108 (40.10)	0.051
Total cholesterol (mmol/L)	4.80 (4.04-5.72)	4.73 (4.06-5.54)	0.599
Triglyceride (mmol/L)	1.56 (1.14-2.44)	1.55 (1.07-2.20)	0.216
LDL cholesterol (mmol/L)	3.12 (2.52-3.82)	3.14 (2.54-3.75)	0.762
HDL cholesterol (mmol/L)	1.08 (0.93-1.32)	1.09 (0.93-1.29)	0.724
Hs-CRP (mg/L)	1.58 (0.87-3.36)	1.87 (0.93-3.48)	0.267
Fasting blood glucose (mmol/L)	11.36 (9.05-13.89)	11.36 (9.26-10.05)	0.834
TyG index	9.60 (9.12-10.04)	9.57 (9.20-9.91)	0.408
Fasting C-peptide (pmol/L)	465.95 (331.26-626.25)	466.00 (344.00-614.75)	0.484
Fasting insulin (pmol/L)	34.44 (22.39-48.07)	33.37 (23.22-49.50)	0.742
HbA1c (%)	10.90 (9.50-12.50)	11.00 (9.20-12.40)	0.663

Data are presented as median (P_25_-P_75_) or number (%).

TyG, triglyceride-glucose; BMI, body mass index; SBP, systolic blood pressure; DBP, diastolic blood pressure; HDL, high density lipoprotein; LDL, low density lipoprotein; hs-CRP, high sensitivity C-reactive protein; HbA1c, haemoglobin. Both * symbol and bold values provided in **Table 1** indicate significant differences between the two groups (P < 0.05).

### Clinical elements: Model 1

In terms of clinical elements, age and smoking history were independent risk factors associated with carotid plaque burden in patients with newly diagnosed diabetes, while age, smoking history, and TyG index were independent risk factors associated with high carotid plaque burden (PS ≥ 3) in patients with newly diagnosed diabetes. The results of the logistic regression analysis are shown in **Table 2**.

**Table 2:** Univariate and multivariate logistic regression analysis of the clinical elements for the patients with plaque burden.

Variables	Patients with plaque burden				Patients with high plaque burden			
	Univariate analysis		Multivariate analysis		Univariate analysis		Multivariate analysis	
	OR (95% CI)	*P*-value	OR (95% CI)	*P*-value	OR (95% CI)	*P*-value	OR (95% CI)	*P*-value
Age	1.0711.042-1.101)	**0.000**	1.065 (1.035-1.096)	**0.000**	1.081 (1.037-1.128)	**0.000**	1.111 (1.060-1.165)	**0.000**
Gender (female vs. male)	1.260 (0.817-1.943)	0.296			1.880 (0.946-3.736)	0.071		
Smoking history (yes vs. no)	1.756 (1.171-2.635)	**0.006**	1.978 (1.285-3.044)	**0.002**	2.040 (1.118-3.723)	**0.020**	2.323 (1.193-4.523)	**0.013**
Drinking history (yes vs. no)	1.338 (0.882-2.029)	0.171			0.898 (0.493-1.633)	0.724		
Hypertension history (yes vs. no)	1.720 (1.146-2.583)	**0.009**	1.18 (0.737-1.888)	0.491	1.811 (0.989-3.315)	0.054		
SBP (mmHg)	1.019 (1.008-1.031)	**0.001**	1.017 (1.004-1.030)	**0.010**	1.009 (0.994-1.025)	0.252		
DBP (mmHg)	1.000 (0.982-1.017)	0.978			1.002 (0.978-1.027)	0.869		
BMI (kg/m^2^)	2.603 (0.914-1.010)	0.120			0.968 (0.891-1.053)	0.452		
Waist circumference (cm)	0.989 (0.969-1.009)	0.261			1.003 (0.975-1.032)	0.846		
Total cholesterol (mmol/L)	0.972 (0.840-1.125)	0.704			1.116 (0.901-1.381)	0.315		
Triglyceride (mmol/L)	0.938 (0.836-1.053)	0.279			1.248 (1.002-1.556)	**0.048**	0.682 (0.303-1.535)	0.355
HDL cholesterol (mmol/L)	0.860 (0.588-1.257)	0.435			0.463 (0.166-1.296)	0.143		
LDL cholesterol (mmol/L)	0.983 (0.921-1.049)	0.600			1.201 (0.865-1.666)	0.274		
Fasting blood glucose (mmol/L)	0.988 (0.932-1.048)	0.686			1.039 (0.951-1.135)	0.395		
Fasting C-peptide (pmol/L)	1.000 (1.000-1.001)	0.409			1.000 (0.998-1.001)	0.673		
Fasting insulin (pmol/L)	0.999 (0.995-1.003)	0.527			0.994 (0.982-1.006)	0.312		
HbA1c (%)	0.967 (0.885-1.058)	0.465			1.059 (0.940-1.193)	0.344		
TyG index	0.852 (0.627-1.157)	0.305			2.167 (1.273-3.688)	**0.004**	3.798 (1.409-10.239)	**0.008**
Hs-CRP (mg/L)	0.947 (0.768-1.169)	0.615			0.996 (0.978-1.015)	0.688		

TyG, triglyceride-glucose; BMI, body mass index; SBP, systolic blood pressure; DBP, diastolic blood pressure; HDL, high density lipoprotein; LDL, low density lipoprotein; hs-CRP, high sensitivity C-reactive protein; HbA1c, haemoglobin; OR, odd ratio; CI, confidence interval. Bold values provided in **Table 2** indicate P < 0.05.

A logistic regression classifier was established according to the selected clinical characteristics. In all subjects included in the study, the AUC of the training set was 0.687 (95% CI: 0.634-0.730), the accuracy rate was 0.656, the sensitivity was 0.617, and the specificity was 0.694. The AUC of the validation set was 0.721 (95% CI: 0.642-0.799), the accuracy rate was 0.685, the sensitivity was 0.691, and the specificity was 0.679. In subjects with a high plaque burden, the AUC of the training set was 0.755 (95% CI: 0.683-0.826), the accuracy rate was 0.697, the sensitivity was 0.629, and the specificity was 0.737. The AUC of the validation set was 0.620 (95% CI: 0.490-0.750), the accuracy rate was 0.654, the sensitivity was 0.633, and the specificity was 0.667.

### Radiomics signature: Model 2

ICCs that represented the intraobserver and interobserver consistency of the feature extraction were calculated. A total of 1270 stable features with ICCs greater than 0.75 were retained for subsequent analysis. After applying the LASSO algorithm in the training set, ten features associated with the present of arteriosclerosis and ten features associated with the severity of arteriosclerosis were selected from the extracted features. These twenty features included eight first-order features and twelve texture features, which can be seen in **Table 3** and **Supplementary Figure S1**. Based on the selected features, the linear regression (LR) algorithm was used to construct the radiomics model and to calculate the Rad-score (Supplementary Appendix S1).

**Table 3:** Radiomics features extracted from chest CT image that were significantly relevant with the present of arteriosclerosis and the severity of arteriosclerosis.

No.	Radiomics features based on chest CT	Coefficients	Relative to max
	**Significantly relevant with the present of arteriosclerosis**		
1	wavelet-HL_glszm_LowGrayLevelZoneEmphasis	0.8349	1
2	wavelet-LH_gldm_LargeDependenceHighGrayLevelEmphasis	0.6438	0.7712
3	wavelet-HH_firstorder_Skewness	0.5016	0.6008
4	square_firstorder_RobustMeanAbsoluteDeviation	0.3553	0.4255
5	wavelet-HH_glrlm_LongRunLowGrayLevelEmphasis	0.3379	0.4048
6	wavelet-HL_glszm_SmallAreaLowGrayLevelEmphasis	0.2434	0.2915
7	wavelet-HL_gldm_SmallDependenceHighGrayLevelEmphasis	-0.4936	-0.5913
8	wavelet-LL_firstorder_Skewness	-0.5673	-0.6795
9	lbp-3D-k_glrlm_RunLengthNonUniformity	-0.6227	-0.7458
10	exponential_glrlm_GrayLevelNonUniformity	-0.7649	-0.9161
	**Significantly relevant with the severity of arteriosclerosis**		
1	wavelet-HL_glrlm_HighGrayLevelRunEmphasis	0.3176	0.5121
2	wavelet-HL_firstorder_Skewness	0.3161	0.5096
3	wavelet-HH_glszm_GrayLevelNonUniformity	0.262	0.4225
4	wavelet-HH_glszm_LowGrayLevelZoneEmphasis	0.2403	0.3874
5	wavelet-HH_glszm_SmallAreaLowGrayLevelEmphasis	0.2116	0.3412
6	gradient_firstorder_Kurtosis	-0.1933	-0.3116
7	square_firstorder_Range	-0.2177	-0.351
8	wavelet-HL_glcm_JointEnergy	-0.2254	-0.3635
9	lbp-3D-k_firstorder_Variance	-0.4229	-0.6818
10	wavelet-HH_firstorder_Median	-0.6202	-1

For all subjects included in the study, the AUC of the training set was 0.932 (0.907-0.957), the accuracy rate was 0.862, the sensitivity was 0.872, and the specificity was 0.852. The AUC of the validation set was 0.927 (0.890-0.964), the accuracy rate was 0.842, the sensitivity was 0.840, and the specificity was 0.845. In subjects with a high plaque burden, the AUC of the training set was 0.734 (0.663-0.805), the accuracy rate was 0.670, the sensitivity was 0.771, and the specificity was 0.610. The AUC of the validation set was 0.698 (0.582-0.814), the accuracy rate was 0.642, the sensitivity was 0.567, and the specificity was 0.686.

### Clinical-radiomics combined model: Model 3

**Figure 3:**
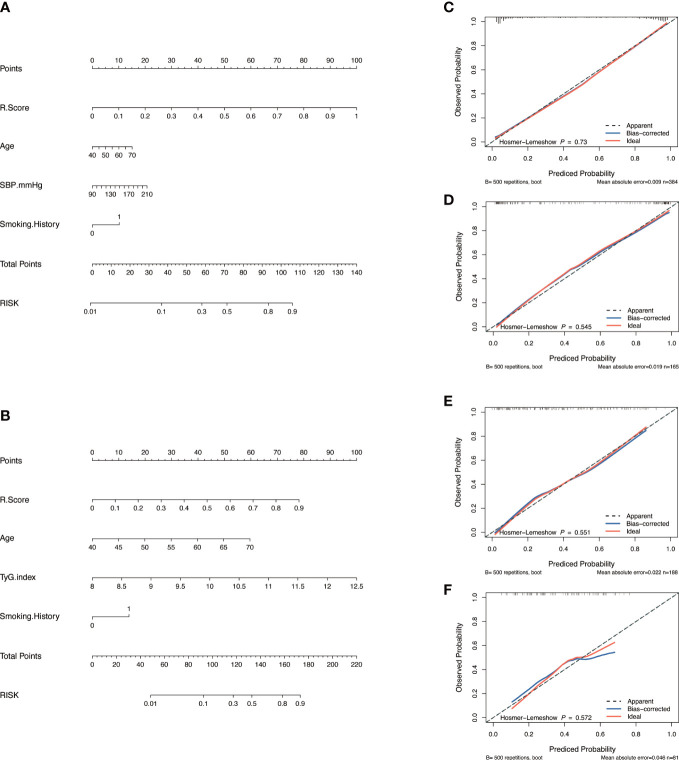
Model construction and evaluation. Nomograms of the combined model to indicate plaque presence **(A)** and plaque severity **(B)**. Calibration curves of the nomogram indicating plaque presence for the training set **(C)** and the validation set **(D)**. Calibration curves of the nomogram indicating plaque severity for the training set **(E)** and the validation set **(F)**. The x-axes represent the probability of plaque presence or severity evaluated by the combined models, and the y-axes represent the actual rate of plaque presence or severity. The diagonal dotted lines represent perfect predictions by ideal models, while the solid lines represent the discrimination abilities of the nomograms, of which closer fits to the diagonal dotted lines represent better evaluations.

**Figure 4:**
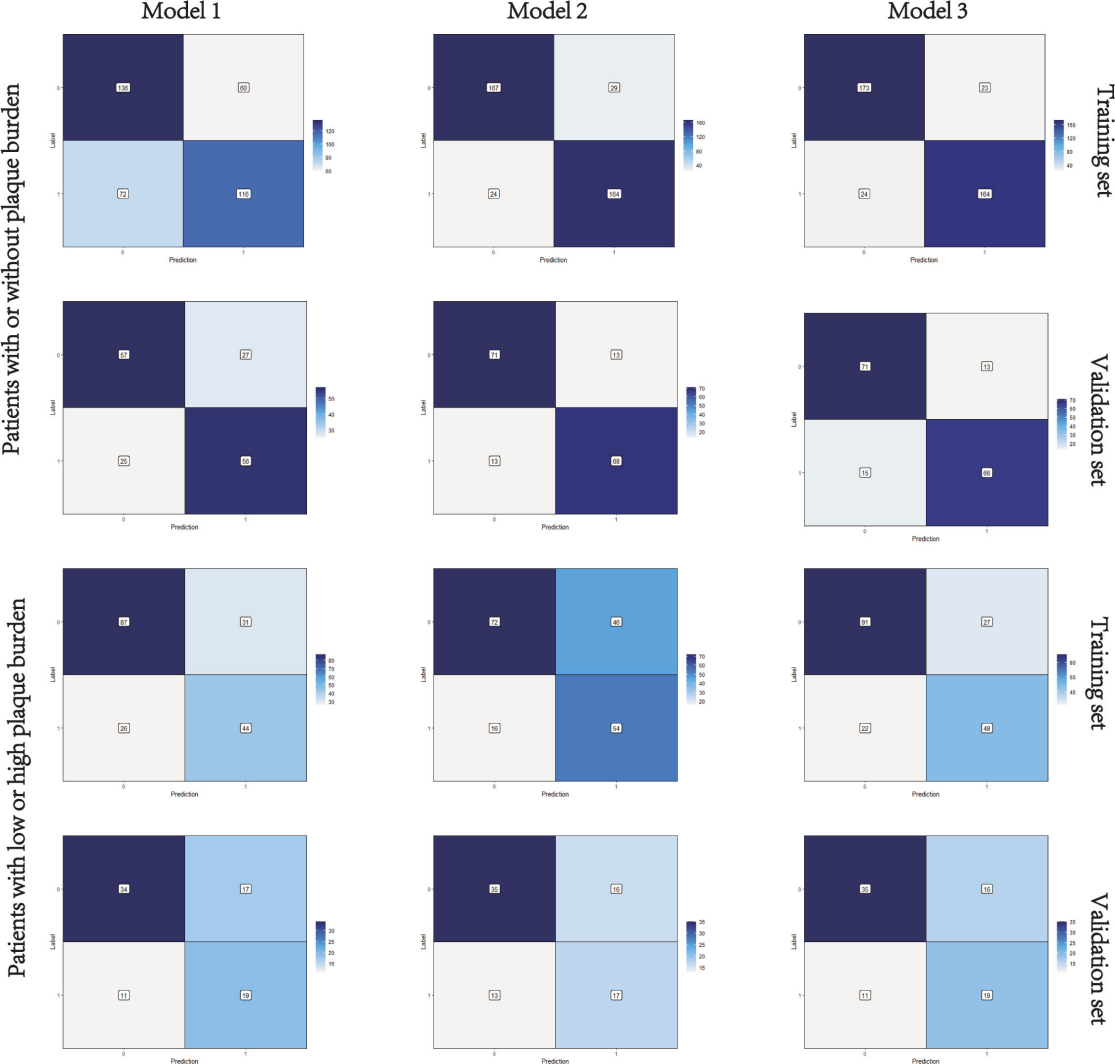
Confusion matrixes of the three models. Model 1, clinical model; Model 2, radiomics model; Model 3, clinical-radiomics combined model. The x-axes represent the predicted labels, and the y-axes represent the actual labels. Four quadrants clockwise from the upper left hand refer to true negatives, false positives, false negatives, and true positives, respectively. The false-positive and false-negative rates in model 3 were lower than model 1 in both the training sets and validation sets.

**Figure 5:**
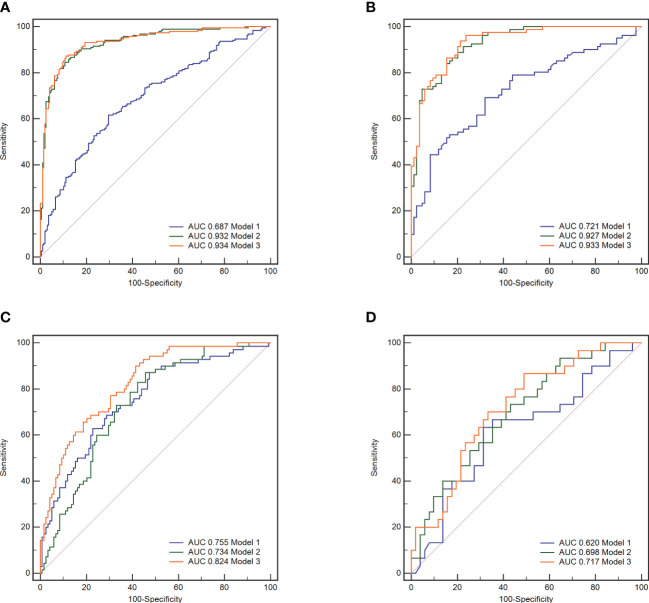
The receiver operating characteristic (ROC) curves of the three models. Model 1, clinical model; Model 2, radiomics model; Model 3, clinical-radiomics combined model. In all subjects included in the study, model 3 demonstrated better indicative efficacy than model 1 and similar to model 2, with an AUC of 0.934 in the training set **(A)** and an AUC of 0.933 in the validation set **(B)**. In subjects with high plaque burden, model 3 demonstrated the best indicative efficacy than the other two models, with an AUC of 0.824 in the training set **(C)** and an AUC of 0.717 in the validation set **(D)**.

Nomograms including clinical factors and Rad-score are shown in **Figure 3**. The formulas to assess the risk probability for atherosclerosis are available in Supplementary Appendix S2. The performances of the three models are presented by a confusion matrix. The false-positive and false-negative rates in model 3 were lower than model 1 in both the training sets and validation sets (**Figure 4**, **Supplementary Table S1**). In all subjects included in the study, the AUC of model 3 was higher than that of model 1 [0.934 (0.909, 0.959) vs. 0.687 (0.634, 0.730), *P* < 0.001 in the training set, 0.933 (0.898, 0.969) vs. 0.721 (0.642, 0.799), *P* < 0.001 in the validation set]. Similar indicative efficacy was found between model 3 and model 2 (*P* = 0.5694). In subjects with high plaque burden, the AUC of model 3 was higher than that of both model 1 and model 2 [0.824 (0.765, 0.882) vs. 0.755 (0.683, 0.826) and 0.734 (0.663, 0.805), *P* < 0.001 in the training set, 0.717 (0.604, 0.830) vs. 0.620 (0.490, 0.750) and 0.698 (0.582, 0.814), *P* < 0.001 in the validation set, respectively]. **Table 4** lists the AUC, accuracy, sensitivity and specificity of the three models. The comparison of the ROC curves of the three models is presented in **Figure 5**.

**Table 4:** The area under curve, accuracy, sensitivity, specificity, negative predictive value, and positive predictive value of the three models.

Model	AUC (95% CI)	Sensitivity	Specificity	ACC	NPV	PPV
Patients with or without plaque burden						
Training set (n=384)						
Model 1	0.687(0.634-0.730)	0.617	0.694	0.656	0.654	0.659
Model 2	0.932(0.907-0.957)	0.872	0.852	0.862	0.874	0.850
Model 3	0.934(0.909-0.959)	0.872	0.883	0.934	0.878	0.877
Validation set (n=165)						
Model 1	0.721(0.642-0.799)	0.691	0.679	0.685	0.695	0.675
Model 2	0.927(0.890-0.964)	0.840	0.845	0.842	0.845	0.840
Model 3	0.933(0.898-0.969)	0.815	0.845	0.830	0.826	0.835
Patients with low or high plaque burden						
Training set (n=188)						
Model 1	0.755(0.683-0.826)	0.629	0.737	0.697	0.770	0.587
Model 2	0.734(0.663-0.805)	0.771	0.610	0.670	0.540	0.818
Model 3	0.824(0.765-0.882)	0.686	0.771	0.739	0.805	0.640
Validation set (n=81)						
Model 1	0.620(0.490-0.750)	0.633	0.667	0.654	0.756	0.528
Model 2	0.698(0.582-0.814)	0.567	0.686	0.642	0.729	0.515
Model 3	0.717(0.604-0.830)	0.633	0.686	0.667	0.761	0.543

AUC, area under curve; CI, confidence interval; ACC, accuracy; NPV, negative predictive value; PPV, positive predictive value. Model 1, clinical model; Model 2, radiomics model; Model 3, clinical-radiomics combined model.

### The performance of the nomogram

**Figure 6:**
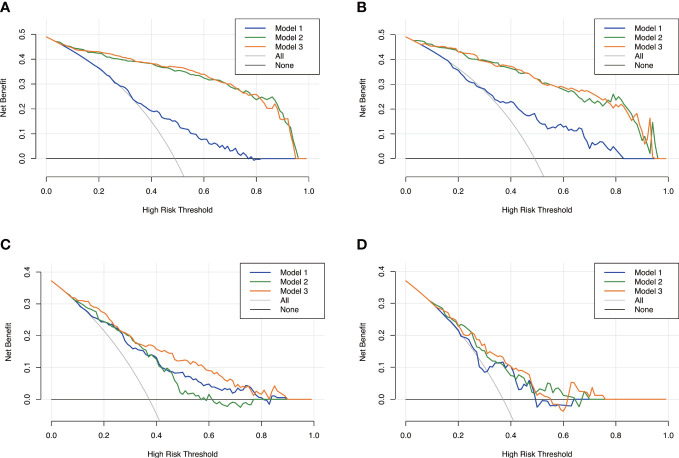
Decision curves analysis for the three models. Model 1, clinical model; Model 2, radiomics model; Model 3, clinical-radiomics combined model. The x-axes represent the threshold probability, and the y- axes represent the net benefit. For all subjects included in the study, model 3 and model 2 had better performance than model 1 in both the training set **(A)** and the validation set **(B)**. However, for subjects with high plaque burden, model 3 had higher efficacy than the other two models in the training set **(C)** and no significant differences in the validation set **(D)**.

The calibration curve along with the H-L test demonstrated good consistency between the observed carotid plaque burden and indicated arteriosclerosis in both the training and validation sets (**Figures 3C–F**). DCA showed that model 3 and model 2 had better performance than model 1 for all subjects included in the study in both training set and validation set, as shown in **Figure 6**. However, for subjects with a high plaque burden, model 3 had higher efficacy than the other two models in the training set and showed no significant differences in the validation set.

## Discussion

In this study, we used radiomics intermuscular adipose analysis as a novel marker to assess arteriosclerosis in patients with newly diagnosed T2D. A radiomics model relating to arteriosclerosis was established by extracting features from medical images and choosing the effective characteristics. A clinical-radiomics combined model was developed by combining the Rad-score with clinical risk factors. Nomograms were then constructed to indicate plaque presence and severity in our study population. The results showed that the combined model improved the accuracy of arteriosclerosis indication both in diagnostic performance and clinical net benefit compared with the model using only clinical risk factors or radiomics features.

Since the risk of arteriosclerosis is present in the state of newly diagnosed T2D, it is critical to choose the subjects at stake for early prevention (22). Multiple studies have revealed that IR is a predictor of arteriosclerosis and could be used to assess the risk of arteriosclerosis in patients with diabetes (23). However, research on the early stage of arteriosclerosis in newly diagnosed T2D is limited (3). TG/HDL-C, TyG, and visceral adiposity index are commonly used as markers for IR identification (24). In our study, no significant differences could be found in either TG/HDL-C or TyG between the patients with or without plaque burden. This may be due to the fact that abnormalities in laboratory tests are less pronounced in the population of newly diagnosed T2D. The diagnostic efficacy of the clinical model constructed by traditional risk factors in indicating arteriosclerosis in patients with newly diagnosed T2D may be limited. To support this viewpoint, another clinical model was constructed in patients with a high carotid plaque burden, and it was found that the TyG index was associated with a higher level of arteriosclerosis. This result indicated that the efficiency of serological markers is more significant in indicating arteriosclerosis extension rather than early arteriosclerosis, which was in agreement with published reports (25, 26). Besides, in our clinical model, only age and smoking history were independent risk factors associated with arteriosclerosis in patients with newly diagnosed diabetes. It may be attributed to clinical stage of our population. Most of the patients included in our analysis were found to have dysglycaemia accidentally during asymptomatic physical examination. In general, the diagnostic efficacy of the clinical model was better in indicating a high level of arteriosclerosis than in indicating the existence of arteriosclerosis in patients with newly diagnosed T2D (0.755 [0.683, 0.826)] vs. 0.687 [0.634, 0.730], *P* < 0.001). Serological measures may be less sensitive, and better indicators are needed.

Our use of radiomics to quantify IMAT is a strength of the current research. IMAT is a type of adipose tissue depot located beneath the fascia and within the muscles (10). It is understudied due to the limited accessibility in the past. With the increasing development of imaging techniques, noninvasive quantification of IMAT has been used in research and healthcare settings (13, 27–29). Tuttle et al. used magnetic resonance imaging to measure IMAT volume in patients with T2D. They found that IMAT volume was correlated with glycated haemoglobin levels and were associated with IR (30). Pishgar et al. used CT to quantify the IMAT area in patients with chronic obstructive pulmonary disease and found that the IMAT area was negatively correlated with lung function (13). Nevertheless, quantifying IMAT by traditional imaging methods is somewhat difficult (11). In our study, we used radiomics to extract texture features that were unrecognized by the naked eye for further quantitative analyses. By applying LASSO and linear regression algorithm, eight first-order features that represented the intensity and distribution of pixels in the ROIs, and twelve texture features that represents the heterogeneity between ROIs, were selected. Although Chen et al. (31) previously reported that morphological features were associated with the degree of the diseases, no morphological features were proven to be associated with arteriosclerosis in our study. The reason may be attributed to the fact that it is the relative value of IMAT rather than the absolute value that is meaningful, which was accordant with previous researches about IMAT analysis (13, 29). Besides, texture features such as GLSZM and GLDM, and wavelet features obtained by wavelet decomposition of the original image, could represent tiny differences in the imaging characteristics in CT images (32, 33). Thus, radiomics IMAT analysis may reflect the internal heterogeneity of the ROIs more accurately.

Three models were constructed to evaluate the risk of arteriosclerosis in our study. By combining radiomics features and clinical risk factors together, we found that the diagnostic performance and clinical net benefit of the combined model in arteriosclerosis indication in newly diagnosed T2D were improved in both the training set and validation set. The combined model leads to an improvement in not only sensitivity (from 0.617 to 0.827 in the training set and from 0.691 to 0.815 in the validation set) but also specificity (from 0.656 to 0.934 in the training set and from 0.685 to 0.830 in the validation set). This result can be explained by the fact that combined model integrated the clinical information and both macro and micro structure characteristics, which could help in further improving the diagnostic efficiency (34). The combined model seems to present excellent value for indicating the presence of plaque burden. However, it is worth noting that in indicating low or high plaque burden, the performance of the combined model increased only slightly, with an AUC improvement from 0.755 to 0.824 in the training set and from 0.620 to 0.717 in the validation set. The reason may be attributable to the fact that in the population with a high level of arteriosclerosis, the indicative value of clinical indicators improved while the role of imaging became less sensitive.

Finally, the nomogram forecast models were applied in clinical practice. The nomogram forecast models are charts with scales that contain varieties of disease risk elements. They can predict the probability of clinical outcomes by using a risk score, which is simpler and easier to understand (35). As seen in our results, the Rad-score was more important in indicating the presence of arteriosclerosis. In indicating a high level of arteriosclerosis, the TyG index held a more prominent position. The nomograms of the combined model offered a more user-friendly way for physicians to identify the risk of arteriosclerosis and could be a convenient method of arteriosclerosis indication in clinical work.

## Limitations

There are several limitations in the current study. First, the study was conducted retrospectively at a single centre. However, the performance of the models was validated through randomization. To provide better evidence for clinical application, multicentre validation with a larger sample size may be necessary. Second, carotid plaque burden evaluated by ultrasound examination was applied as the marker of arteriosclerosis, which may not be as accurate as the pathologic biopsy in representing arteriosclerosis. However, it has been reported that the prevalence of carotid artery plaques could well reflect the overall severity of arteriosclerosis in the vasculature (36). Third, radiomics features were only extracted from a single section but not the whole body. Quantitative analysis of IMAT within the whole body is indeed more precise but more time-consuming and has a higher requirement for equipment. Fourth, the efficacy of IMAT analysis in indicating arteriosclerosis in subjects without T2DM was not conducted in the current study. Our results imply that the radiomics IMAT analysis derived from the section above the aortic arch could be a novel marker to assess the degree of arteriosclerosis, which may provide a more convenient way to evaluate early arteriosclerosis in patients with newly diagnosed T2D. Finally, in the process of radiomics feature recognition, two radiologists took several times to manually delineated the borders of the pectoralis major muscle. Future studies could improve efficiency and accuracy by using efficient automatic segmentation.

## Conclusions

Radiomics intermuscular adipose analysis could indicate the present and severity of arteriosclerosis, providing a novel marker for the assessment of arteriosclerosis in patients with newly diagnosed T2D. Though radiomic analysis, features about intensity and distribution of pixels as well as features about texture were selected for the quantification. The clinical-radiomics combined model showed great performance and high sensitivity in indicating arteriosclerosis. Moreover, the constructed nomograms could provide a quantitative and intuitive way to indicate arteriosclerosis, which may help clinicians comprehensively analyse radiomics characteristics and clinical risk factors more confidently.

## Data availability statement

The raw data supporting the conclusions of this article will be made available by the authors, without undue reservation.

## Ethics statement

The studies involving human participants were reviewed and approved by Ethics Committee of Shaoxing Second Hospital. The ethics committee waived the requirement of written informed consent for participation.

## Author contributions

CH were responsible for conceptualization, investigation and writing the original draft. DX, L-FF and J-NY contributed to image interpretation and data collection. F-YW and Y-GQ contributed to the collection of clinical cases. H-WX contributed to critically revising the manuscript. All authors contributed to the article and approved the submitted version.
