# Supplementary material for: A nomogram based on radiomics intermuscular adipose analysis to indicate arteriosclerosis in patients with newly diagnosed type 2 diabetes
Source: Front Endocrinol (Lausanne). 2023 May 26;14:1201110. doi: 10.3389/fendo.2023.1201110 (PMC10250635; doi:10.3389/fendo.2023.1201110)
Supplement: Supplementary file 1 [file DataSheet_1.pdf]

A nomogram based on radiomics intermuscular adipose analysis to indicate arteriosclerosis in patients with newly diagnosed type 2 diabetes

I. **Supplementary Figure S1**

II. **Supplementary Appendix S1**

III. **Supplementary Appendix S2**

IV. **Supplementary Table S1**

(A)

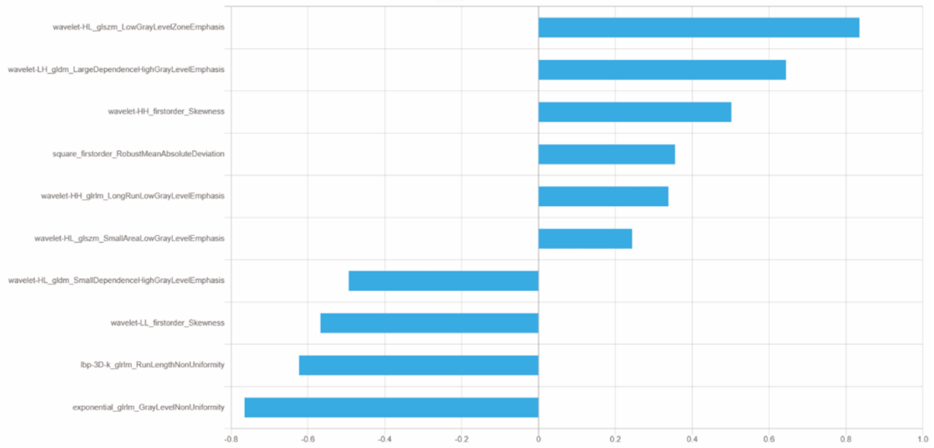

(B)

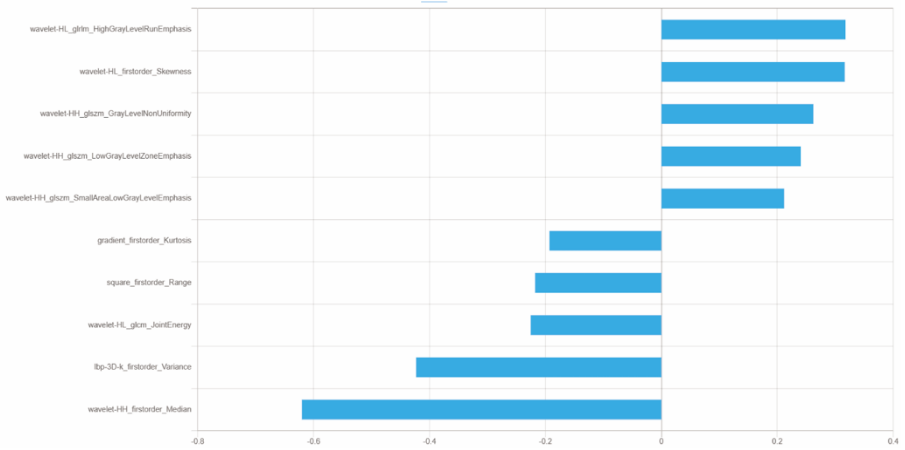

Supplementary Figure S1. Radiomics features extracted from chest CT image that were significantly relevant with the present of arteriosclerosis (A) and severity of arteriosclerosis (B).

Supplementary Appendix S1. Formulas of the radiomics models indicating the present and severity of arteriosclerosis.

Logit (Rad-score $_{present\ of\ arteriosclerosis}$*)* = 0.6438 × wavelet-LH_gldm_LargeDependenceHighGrayLevelEmphasis + 0.8349 × wavelet-HL_glszm_LowGrayLevelZoneEmphasis - 0.4936 × wavelet-HL_gldm_SmallDependenceHighGrayLevelEmphasis + 0.3379 × wavelet-HH_glrlm_LongRunLowGrayLevelEmphasis - 0.7649 × exponential_glrlm_GrayLevelNonUniformity - 0.6227 × lbp-3D-k_glrlm_RunLengthNonUniformity + 0.2434 × wavelet-HL_glszm_SmallAreaLowGrayLevelEmphasis + 0.3553 × square_firstorder_RobustMeanAbsoluteDeviation - 0.5673 × wavelet-LL_firstorder_Skewness + 0.5016 × wavelet-HH_firstorder_Skewness - 0.3526

Logit (Rad-score $_{severity\ of\ arteriosclerosis}$*)*= - 0.6202 × wavelet-HH_firstorder_Median + 0.3176 × wavelet-HL_glrlm_HighGrayLevelRunEmphasis - 0.2177 × square_firstorder_Range + 0.2620 × wavelet-HH_glszm_GrayLevelNonUniformity + 0.3161 × wavelet-HL_firstorder_Skewness - 0.4229 × lbp-3D-k_firstorder_Variance - 0.1933 × gradient_firstorder_Kurtosis + 0.2403 × wavelet-HH_glszm_LowGrayLevelZoneEmphasis + 0.2116 × wavelet-HH_glszm_SmallAreaLowGrayLevelEmphasis - 0.2254 × wavelet-HL_glcm_JointEnergy -0.1514

Supplementary Appendix S2. Formulas to assess the risk probability for the present and severity of atherosclerosis.

$Logit\ (Risk_{present\ of\ atherosclerosis})$ = 2.2645 × Rad-score + 0.2490 × Age + 0.1974 × SBP (mmHg) + 0.3062 × Smoking history (0/1) - 0.0827

$Logit\ (Risk_{severity\ of\ atherosclerosis})$ = logit = 0.9634 × Rad-score + 0.7495 × Age + 0.6629 × TyG index + 0.3950 × Smoking history (0/1) - 0.2535

Supplementary Table S1. The true negatives rate, false positives rate, false negatives rate, and true positives rate of the three models.

Model	TNR	FPR	FNR	TPR
	**Patients with or without plaque burden**			
**Training set (n=384)**				
Model 1	136/196 (69.39%)	60/196 (30.61%)	72/188 (38.30%)	116/188 (61.70%)
Model 2	167/196 (85.20%)	29/196 (14.80%)	24/188 (12.77%)	164/188 (87.23%)
Model 3	173/196 (88.27%)	23/196 (11.73%)	24/188 (12.77%)	164/188 (87.23%)
**Validation set (n=165)**				
Model 1	57/84 (67.86%)	27/84 (32.14%)	25/81 (30.86%)	56/81 (69.14%)
Model 2	71/84 (84.52%)	13/84 (15.48%)	13/81 (16.05%)	68/81 (83.95%)
Model 3	71/84 (84.52%)	13/84 (15.48%)	15/81 (18.52%)	66/81 (81.48%)
	**Patients with low or high plaque burden**			
**Training set (n=188)**				
Model 1	87/118 (73.73%)	31/118 (26.27%)	26/70 (37.14%)	44/70 (62.86%)
Model 2	72/118 (61.02%)	46/118 (38.98%)	16/70 (22.86%)	54/70 (77.14%)
Model 3	91/118 (77.12%)	27/118 (22.88%)	22/70 (31.43%)	48/70 (68.57%)
**Validation set (n=81)**				
Model 1	34/51 (66.67%)	17/51 (33.33%)	11/30 (36.67%)	19/30 (63.33%)
Model 2	35/51 (68.63%)	16/51 (31.37%)	13/30 (43.33%)	17/30 (56.67%)
Model 3	35/51 (68.83%)	16/51 (31.37%)	11/30 (36.67%)	19/30 (63.33%)

TNR, true-negative rate; FPR, false-positive rate; FNR, false-negatives rate; TPR, true-positives rate; Model 1, clinical model; Model 2, radiomics model; Model 3, clinical-radiomics combined model.
